# Methylation Status of Vitamin D Receptor Gene Promoter in Benign and Malignant Adrenal Tumors

**DOI:** 10.1155/2015/375349

**Published:** 2015-12-30

**Authors:** Catia Pilon, Andrea Rebellato, Riccardo Urbanet, Vincenza Guzzardo, Rocco Cappellesso, Hironobu Sasano, Ambrogio Fassina, Francesco Fallo

**Affiliations:** ^1^Clinica Medica 3, Department of Medicine-DIMED, University of Padova, 35128 Padova, Italy; ^2^Cytopathology Unit, Department of Medicine-DIMED, University of Padova, 35128 Padova, Italy; ^3^Department of Pathology, Tohoku University Graduate School of Medicine, Sendai 980-8575, Japan

## Abstract

We previously showed a decreased expression of vitamin D receptor (VDR) mRNA/protein in a small group of adrenocortical carcinoma (ACC) tissues, suggesting the loss of a protective role of VDR against malignant cell growth in this cancer type. Downregulation of VDR gene expression may result from epigenetics events, that is, methylation of cytosine nucleotide of CpG islands in VDR gene promoter. We analyzed methylation of CpG sites in the VDR gene promoter in normal adrenals and adrenocortical tumor samples. Methylation of CpG-rich 5′ regions was assessed by bisulfite sequencing PCR using bisulfite-treated DNA from archival microdissected paraffin-embedded adrenocortical tissues. Three normal adrenals and 23 various adrenocortical tumor samples (15 adenomas and 8 carcinomas) were studied. Methylation in the promoter region of VDR gene was found in 3/8 ACCs, while no VDR gene methylation was observed in normal adrenals and adrenocortical adenomas. VDR mRNA and protein levels were lower in ACCs than in benign tumors, and VDR immunostaining was weak or negative in ACCs, including all 3 methylated tissue samples. The association between VDR gene promoter methylation and reduced VDR gene expression is not a rare event in ACC, suggesting that VDR epigenetic inactivation may have a role in adrenocortical carcinogenesis.

## 1. Introduction

Besides the classical role in calcium and bone homeostasis, 1*α*,25-dihydroxycholecalciferol D3 [1*α*,25(OH)_2_D_3_] (calcitriol), the active metabolite of vitamin D, has been recognized to have “noncalcemic” effects in a variety of cells after binding to vitamin D receptor (VDR, NR1I1), a member of the nuclear receptor superfamily which includes receptors for steroids, thyroid hormones, and retinoic acid [1]. The VDR forms homodimers or heterodimers with the retinoid X receptor (RXR, NR2B), to allow specific DNA binding. The binding of 1*α*,25(OH)_2_D_3_ with VDR-RXR complex is followed by the attachment of this complex to vitamin D responsive elements, which then initiate transcription in the promoter of target genes [2, 3]. The effect of liganded VDR depends on the epigenetic landscape of target gene [4].

There is evidence that 1*α*,25(OH)_2_D_3_ protects against tumor formation by several VDR-mediated mechanisms, including regulation of growth arrest, cell differentiation, migration, invasion, and apoptosis, making it a candidate agent for cancer regulation [5–7]. A relationship between the vitamin D system and the adrenal pathophysiology and growth has been recently highlighted [8]. We showed a decreased expression of VDR mRNA and protein in a small group of human adrenocortical carcinomas (ACCs), suggesting the loss of a protective role of VDR against malignant cell growth, as suggested for other cancer types [9, 10].

An aberrant global and gene-specific DNA promoter methylation has been observed in human adrenocortical tumors, either benign or malignant, implicating dysregulation of steroid biosynthesis and adrenal growth [11–15]. Downregulation of VDR gene expression in adrenal carcinomas may result from epigenetic events, that is, methylation of cytosine nucleotides in CpG island of VDR promoter. In fact, promoter methylation is able to distort the transcription factor binding sites, causing transcriptional silencing. 

In continuity with our previous observations [10], the aim of our study was to analyze methylation of GpG sites in the VDR gene promoter of a different and larger series of human adrenocortical tissues, comparing adrenocortical adenomas (ACAs) with ACCs samples.

## 2. Materials and Methods

### 2.1. Patients and Tissue Samples

This study was approved by the institutional review board of the University Hospital of Padova in accordance with the Declaration of Helsinki guidelines as revised in 1983. Informed consent was obtained from all individual participants included in the study. The preoperative diagnosis was based on the clinical history, symptoms, signs, endocrine evaluation, and imaging examination (e.g., MRI, CT). Archival microdissected paraffin-embedded slides of the patients were used for histological examinations and molecular studies. Diagnosis of adrenal malignancy was performed according to the histopathological criteria proposed by Weiss et al. [16] and the modification proposed by Aubert et al. [17]. Three normal adrenal cortices from adrenal glands of kidney donors were also studied. Histopathological slides were classified by two pathologists (R.C. and A.F.) independently, and no discrepancy existed between them.

### 2.2. VDR Promoter Methylation Analysis

Total genomic DNA was extracted from formalin-fixed paraffin embedded (FFPE) adrenocortical tissues using QIAamp DNA FFPE Tissue Kit (Qiagen, Milan, Italy). DNA samples underwent bisulfite conversion using EZ DNA Methylation-Gold Kit (Zymo Research Co., Milan, Italy). Bisulfite treatment produces a chemical conversion of unmethylated cytosine to uracil, which is detected as a thymine after PCR.

Methylated cytosines are protected from chemical conversion. Bisulfite-treated DNA was amplified using two sets of bisulfite sequencing primers designed by using MethPrimer (http://www.urogene.org/methprimer/index1.html) encompassing the region from −693 bp to −65 bp upstream VDR transcription start site.

Primer sequences are as follows:

  M1F 5′-GGAATTCGGGATTAGGGATTAGGGAAG-3′.
  M1R 5′-AATACGTCACCCCCACCTAAACTAACCAAAC-3′.
  M2F 5′-GTTAGTCGCTAGGCGTTTTTTAGCGTTTCGC-3′.
  M2R 5′-TATAAAACAAAATTATCGATAATTATAAATA-3′.
  M3F 5′-GTAGAATTACGGTAGGAAGGGTGGGGGGTTG-3′.
  M3R 5′-CCCCGCCCACAAATCCAATCCTCTCTTAGG-3′.

PCR products were separated by electrophoresis and isolated using a Centri-Sep Columns (Princeton Separations, Milan, Italy). DNA was sequenced using the Reverse Primer (M1R and M2R) with an Applied Biosystems automated fluorescent sequencer (Applied Biosystems, Milan, Italy). In DNA sequence, methylated sites were visually counted.

### 2.3. RNA Isolation/Quantitative Real-Time PCR (qPCR)

Total cellular RNA was extracted from FFPE adrenal tissue slide samples using RNeasy Universal kit (Qiagen, Gaithersburg, MD). Briefly, FFPE tissue was deparaffinized and treated with proteinase K, and genomic DNA was removed for total RNA extraction. Total RNA was quantified by NanoDrop 1000 Spectrometer (ThermoScientific, Wilmington, DE). Quality of RNA was analyzed by Agilent Bioanalyzer 2100 (Agilent Technologies, Palo Alto, CA). Evaluation of gene expression was performed by quantitative RT-PCR, as previously described in our recent publication [10]. Quantitative PCR for VDR and the housekeeping HMBS (hydroxymethylbilane synthase) gene primers were as follows: 5′-GAAGCCTTTGGGTCTGAAGTG-3′ (VDR forward), 5′-CCGCCATTGCCTCCATCC-3′ (VDR reverse) and 5′-GGCAATGCGGCTGCAA-3′ (HMBS forward), 5′-GGGTACCCACGCGAATCAC-3′ (HMBS reverse). The annealing temperature was 60°C for all genes. PCR was carried out using a DNA Engine (Opticon 2 continuous fluorescence detection system, MJ Research, Waltham, MA, USA). For each sample, results were normalized with the HMBS rRNA.

### 2.4. Western Blot and Densitometric Analysis

Adrenal tissue slides were deparaffinized using Xylene (Sigma) for 3 × 10 min and protein was extracted using Qproteome FFPE Tissue kit (Qiagen, Milan, Italy) and slides were subjected to western blot analyses by 10% Tris-HCl polyacrylamide gel electrophoresis (PAGE) (Invitrogen Co., Eugene, OR, USA) in running buffer (Tris/Glycine/SDS). Membranes were probed at 4°C overnight with anti-VDR mouse polyclonal antibody (1 : 500, VDR-D6, Santa Cruz Biotechnology, Inc., Santa Cruz, CA, USA) and mouse *β*-actin, Clone AC-15 (1 : 10.000) (Sigma–Aldrich, St. Louis, MO, USA). Primary anti-VDR and anti-*β*-actin antibodies were detected with a secondary goat anti-mouse fluorescent antibody (IRDye 800CW, Li-Cor Biosciences, Milan, Italy) (1 : 15.000). Signal was acquired by Li-Cor Odyssey Clx (Li-Cor Biosciences). Quantification of individual protein bands was measured by Li-Cor Image Studio Digits. For each sample, results were normalized with the housekeeping protein *β*-actin.

### 2.5. Immunohistochemistry

Immunostaining for VDR protein expression was performed in formalin-fixed paraffin-embedded adrenal tissue slides, as described in our recent publication [10]. Immunostaining was performed by the streptavidin-biotin amplification method using a Histfine Kit (Nichirei Co. Ltd., Tokyo, Japan). Antigen retrieval was performed by heating the slides in an autoclave for 5 min in citric acid buffer (2 mM citric acid and 9 mM trisodium citrate dehydrate, pH 6.0). The dilution of the primary antibodies was 1 : 50. The antigen-antibody complex was visualized with 3,3′-diaminobenzidine solution [1 mM 3,3′-diaminobenzidine, 50 mM Tris-HCl buffer (pH 7.6), and 0.006% H_2_O_2_] and counterstained with hematoxylin. A human breast cancer specimen was used as a positive control. Negative controls were incubated with normal mouse antiserum instead of the primary antibody, which uniformly demonstrated no reaction (not shown).

### 2.6. Statistics

For two-sample comparison, differences between means were assessed by Mann-Whitney *U* test. Relationships between continuous variables were assessed calculating Spearman's rank correlation coefficient. All results are expressed as mean ± SD for continuous variables. *P* values <0.05 were taken as statistically significant. Statistical analysis was performed using the GraphPad Prism version 6.0 software (GraphPad Software).

## 3. Results

Twenty-three patients (12 females, 11 males) who underwent adrenalectomy for sporadic adrenocortical tumors between 2006 and 2014 were classified as ACAs (*n* = 15) and ACCs (*n* = 8). The cohort was different from that described in our previous publication [10]. Fifteen patients with ACA included 2 cortisol-producing adenomas, 10 aldosterone-producing adenomas, and 3 nonfunctioning adenomas: 7 were females and 8 males, ranging from 31 to 67 years of age (mean age of 51.3 years at presentation). Eight ACCs consisted of 5 females and 3 males, ranging from 33 to 73 years of age (mean age of 52.7 years).

Clinical and tumor characteristics of the 8 ACC patients, including ENSAT stage at surgery [18], are shown in Table 1. Five ACCs patients had endocrine symptoms and signs of excess cortisol secretion; three patients had nonfunctioning adrenal mass. All patients with ACCs were treated with the adrenolytic drug mitotane, 1,1-dichloro-2-(o-chlorophenyl)-2-(p-chlorophenyl) ethane (o,p′-DDD), before surgery. Specifically, mitotane was given before surgery to the five patients with Cushing's syndrome because of hypercortisolism not amenable by other inhibitors of steroidogenesis; the remaining 3 patients were treated with mitotane as adjuvant therapy before second operation for recurrent disease. Mean adrenal tumor diameter in ACAs and ACCs group was 14 mm and 120 mm, respectively. Mean postsurgery follow-up of patients was 72 months (range of 12–120 months) for ACAs and 26 months (range of 6–48 months) for ACCs.

**Figure 1:**
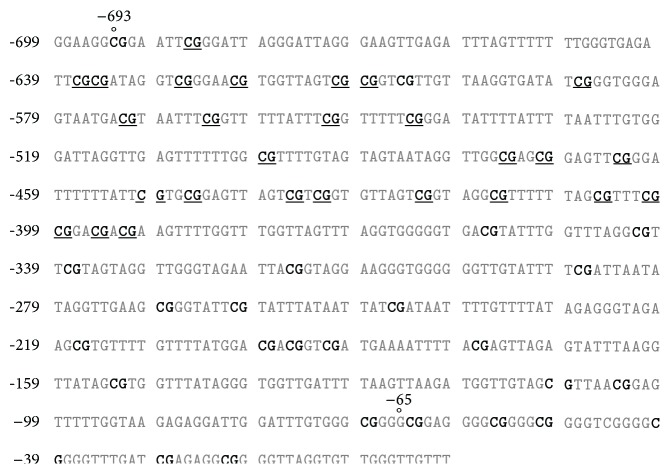
VDR methylated sites of VDR promoter region from −693 to −65 bp in one representative adrenocortical carcinoma, analyzed by bisulfite sequencing. In DNA sequence, bold types indicate all CpG dinucleotides, and underlined CpG indicate methylated sites.

Methylation in the VDR promoter was observed in 3/8 ACCs specimens, which included two cortisol-producing and 1 nonfunctioning carcinoma patients (C3, C4, and C6 patients in Table 1). Two PCR products, including region from −693 to −65 bp, contained 42 CpG islands, and 27 of them (64%) were methylated. One representative case is presented in Figure 1. Methylation sites were identical in all 3 ACCs tissue specimens. No VDR promoter methylation was found in the other 5 ACCs, 3 normal adrenals, and the 15 ACAs.

**Figure 2:**
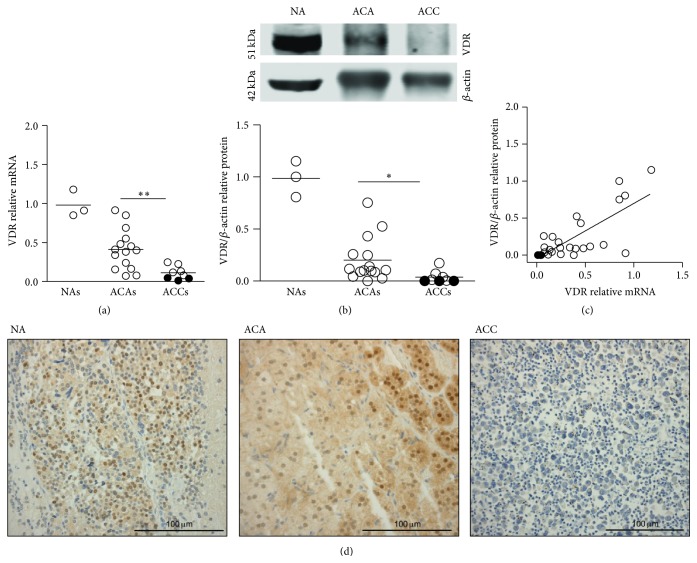
Expression of VDR in normal human adrenals, in adrenocortical adenomas (ACAs), and in adrenocortical carcinomas (ACCs). (a) Individual mRNA levels and means (horizontal bars) of VDR, measured by qPCR, in normal adrenals (*n* = 3), ACAs (*n* = 15), and ACCs (*n* = 8). (b) VDR immunoblot (above) in representative cases of a normal adrenal (NA), ACAs, and ACCs, and individual VDR/*β*-actin protein levels (below) in normal adrenals, ACAs, and ACCs. (c) Correlation between VDR mRNA and VDR protein levels in all tissue samples, including normal and neoplastic adrenal. (d) Immunohistochemical staining of VDR in a normal adrenal (left panel), one ACA (central panel), and one methylated ACC (right panel), showing clear VDR expression in NA, in a cortisol-producing ACA, in both the nucleus and predominantly the cytoplasm, and very weak VDR expression, limited to rare cells, in a cortisol-producing ACC. Sections were counterstained with hematoxylin. Black dots indicate methylated tissue samples. Mann-Whitney *t*-test ACAs versus ACCs. ^*∗∗*^ *P* < 0.01; ^*∗*^ *P* < 0.05. Spearman correlation *r*_*s*_ = 0.56; *P* < 0.003.

qPCR analysis demonstrated variable levels of VDR mRNA in all adrenal tumors, with VDRmRNA expressed at higher levels in ACAs than in ACCs (0.41 ± 0.2 versus 0.11 ± 0.08 arbitrary units, *P* < 0.01) (Figure 2(a)). VDR immunoblot in representative cases of a normal adrenal (NA), ACAs, and ACCs is shown in Figure 2(b). VDR/*β*-actin protein levels, measured in the entire series of tumor specimens, showed results similar to VDR mRNA in terms of difference between benign and malignant tumors (0.20 ± 0.2 versus 0.04 ± 0.06 arbitrary units, *P* < 0.05) (Figure 2(b)). Low or absent VDR expression was observed in individual cases of either ACAs or in ACCs. A positive correlation between VDR mRNA and VDR/*β*-actin levels (*P* < 0.003) was observed (Figure 2(c)).

Immunohistochemical staining for VDR of representative cases of one normal adrenal, one ACA, and one of the 3 methylated ACCs is reported in Figure 2(d). Both nuclear and cytoplasmic VDR immunostaining, consistent with translocation of VDR from cytoplasm to the nucleus after ligand binding [10], were observed in the 3 normal adrenals and in ACAs. At variance, expression of VDR was undetectable or very weak and limited to only scattered tumor cells in all ACCs, including the 3 methylated cases (Figure 2(d)).

## 4. Discussion

The reduced-absent expression of VDR mRNA and protein in adrenocortical cancer may be caused by different molecular mechanism. A somatic VDR gene mutation could occur, reflecting a mechanism (i.e., loss of tumor-suppressor function) implicated in the malignant transformation of adrenocortical cells. We did not analyze this possibility in our samples, but VDR gene is rarely mutated during carcinogenesis [19]. No evidence for VDR gene mutation in a recent whole-exome sequencing analysis of a very large number of ACCs [20] makes however unlikely this event. Epigenetic inactivation of human VDR, reducing its mRNA and protein expression, has been shown in various cancer types [4, 21], supporting the loss of an antiproliferative role of VDR [10], and may potentially occur in ACCs. The promoter of VDR gene lies in a GC-rich island and contains strong regulatory elements for its transcriptional activity [22]. Disruption of promoter activity by DNA methylation is an epigenetic inactivating mechanism frequently observed in tumor-suppressor genes [23]. Our present results support a VDR silencing through this epigenetic mechanism in a subgroup of ACCs. Since a growing body of evidence indicates that DNA promoter methylation can be a consequence rather than a cause of transcriptional inactivation, the hypothesis of VDR methylation as the result of malignant transformation cannot be also excluded. Moreover, methylation of VDR promoter has not been specifically reported in all genome-wide methylation studies on adrenocortical cancer [12–14].

Different epigenetic mechanisms explaining the downregulation of VDR gene expression can also be hypothesized for our remaining ACCs and for some of our adenoma cases with low VDR expression. Altered activity of regulatory elements of VDR transcriptional activity, such as repressors/corepressors or unbalanced enhancers, could be considered [4, 24]. Furthermore, the VDR gene promoter contains an array of putative binding sites for transcription factors mediating the activities of PKA and PKC pathways [22] that are in turn known to converge on several specific transcription factors. The mechanism of action of the liganded VDR is also dependent on a plethora of enzymes regulating covalent histone modifications, and this epigenetic regulatory system has been found frequently altered in cancer.

We cannot exclude that low expression of VDR gene in adrenocortical cancers, as well as in some benign adenomas, may rather be due to the effect of hormonal compounds, that is, estrogens, thyroid hormone, and glucocorticoids, which are likewise able to alter VDR mRNA/protein levels [3]. Interestingly, we showed a critical role of estrogens and ER*α* in adrenal tumorigenesis [25]. Moreover, mitotane, the drug used for treatment of all our ACC patients, is known to stimulate CYP3A4 expression, potentially leading to reduced 1*α*,25(OH)_2_D_3_ bioavailability and reduced VDR expression in adrenals [10]. There is also evidence that a number of short noncoding RNAs may repress VDR posttranscriptional regulation in cancer [26], and their specific role as VDR regulators in adrenocortical tumors is possible [27].

## 5. Conclusions

The main limitation of our study is the relatively small number of samples, and a larger ACCs study population is needed to confirm our results. The study could be enlarged using the adrenal tissue bank of ENSAT (European Network on Adrenal Tumors) collaborative group, which is dedicated to the study and treatment of adrenal tumors, providing study projects and enrolling research teams on this disease. However, our findings represent the first evidence of an association between VDR gene promoter methylation and reduced VDR expression in ACC. This suggests a potential role of VDR epigenetic inactivation in malignant adrenocortical tumorigenesis. Adrenocortical carcinoma, either silent or hormonally active, is a rare tumor with a very poor prognosis, linked to its highly invasive phenotype and marked resistance to radio- and chemotherapy [28]. The VDR promoter methylation might be a target for pharmacological agents to treat adrenal cancer in selected cases [29]. In this regard, the human adrenocortical carcinoma H295R cell line, which provides the most appropriate model for ACC study [30], does not have VDR gene methylation (personal observation). The availability of adrenal cell models allowing the* in vitro* use of DNA methylation inhibitors should be addressed.

## Figures and Tables

**Table 1:** Clinical and tumor characteristics of the ACC patients analyzed in this study.

Sample ID	Age	Gender	Stage at surgery (ENSAT)	Hormonal hypersecretion	Weiss score	Size (mm)	Outcome
C1	58	M	III	Cortisol	6	180	Died for recurrence 4 years after surgery
C2	51	F	III	Cortisol	5	90	Alive, with recurrence
C3	36	M	III	Cortisol	9	110	Died for recurrence 6 months after surgery
C4	73	M	III	Nonfunctioning	9	150	Died for recurrence 1 year after surgery
C5	52	F	IV	Nonfunctioning	10	150	Died for recurrence 2 years after surgery
C6	33	F	III	Cortisol	8	140	Alive, with recurrence
C7	51	M	III	Nonfunctioning	6	60	Alive, with recurrence
C8	68	M	II	Cortisol	9	80	Died for recurrence 2 years after surgery
